# The impact of classic Hodgkin lymphoma on informal caregivers: results from the CONNECT cross-sectional survey

**DOI:** 10.1007/s00520-023-08120-8

**Published:** 2023-11-02

**Authors:** Darcy R. Flora, Andrew M. Evens, Nicholas Liu, Kristina S. Yu, Rachel Byrd, Michelle A. Fanale, Katherine Holmes, Carlos Flores, Andy Surinach, Susan K. Parsons

**Affiliations:** 1 Gryt Health, Rochester, NY USA; 2 Rutgers Cancer Institute of New Jersey, New Brunswick, NJ USA; 3 Seagen Inc., Bothell, WA USA; 4 Ipsos Healthcare, New York, NY USA; 5 Genesis Research, Hoboken, NJ USA; 6 Tufts Medical Center, 800 Washington Street, Boston, MA 02111 USA

**Keywords:** Classic Hodgkin lymphoma, Caregiver, Health-related quality of life, Productivity, Cross-sectional survey

## Abstract

**Purpose:**

As part of the CONNECT study, we evaluated the caregiver role in treatment decision-making when caring for patients with classic Hodgkin lymphoma (cHL) in the USA.

**Methods:**

The CONNECT caregiver survey was administered online December 2020–March 2021 to self-identified adult caregivers of cHL patients recruited from patient referrals and online panels. The caregiver’s role in treatment decision-making, health-related quality of life (HRQoL, PROMIS-Global), and work impacts (WPAI:CG) were assessed.

**Results:**

We surveyed 209 caregivers (58% women; median age 47 years; 54% employed; 53% spouse/partner); 69% of patients cared for were diagnosed with cHL in the past 1–2 years, with 48% having stage III/IV cHL and 29% in remission. More spouse/partner than other caregivers were involved in caregiving at symptom onset (61% vs 27%), whereas more other than spouse/partner caregivers began after first treatment (34% vs 5%). Cure, caregivers’ top treatment goal (49%), was rated higher by spouse/partner than other caregivers (56% vs 42%). More spouse/partner than other caregivers were involved in treatment option discussions with physicians (52% vs 28%), were involved in patients’ treatment decisions (54% vs 23%), and were aligned with patients’ treatment goals (93% vs 79%). While caregivers reported HRQoL similar to that of the general population, nearly 30% of employed caregivers reported work impairment.

**Conclusion:**

Cure was caregivers’ top treatment goal. Spouse/partner vs other caregivers were more involved, were involved earlier, and reported greater alignment with patient treatment goals and decision-making. Caregivers reported good HRQoL; however, caregiving impacted work productivity regardless of patient relationship.

## Introduction

The National Alliance for Caregiving defines a cancer caregiver as someone who, informally and without monetary compensation, cares for a family member or friend diagnosed with cancer [1]. In the USA, approximately 3 to 6 million adults are cancer caregivers, spending an estimated 33 h per week on caregiving activities [1, 2]. Most cancer caregivers (82%) report communicating with the cared-for patient’s health providers [1]. As part of these interactions, caregivers may aid patients in making treatment decisions, with up to 95% of patients with cancer preferring to involve a caregiver in medical decision-making [1]. Positive impacts for both patients and caregivers have been reported when caregivers aid patients in treatment decision-making, including improved adherence to treatment, increased understanding of disease information, greater satisfaction and self-efficacy, and less stress and depression [3–7].

Substantial emotional and financial stress are reported by cancer caregivers, with 50% of these caregivers reporting high levels of emotional stress and 25% of caregivers reporting high levels of financial strain [1]. Caregivers who are employed may require work accommodations to provide care, including different start and stop times, time off, reduced working hours, or a leave of absence [1].

As the trajectory of a cancer diagnosis differs depending on the type of cancer and the disease stage at diagnosis, the burden of caregiving may also differ across cancer diagnoses, as some cancers are associated with a rapid deterioration of the patient’s health, whereas others have high survival rates but a risk of recurrence or a second cancer [1]. Hodgkin lymphoma (HL) is an example of a cancer with high survival rates; among patients diagnosed with it in the USA, the 5-year relative survival rate is 89%, ranging from 81% to 95% depending on disease stage [8]. While many patients with classic HL (cHL) who receive treatment can experience long-term survival, there is a risk of acute and delayed toxicity, including pulmonary toxicity, infertility, second cancers, and cardiovascular effects following treatment with some recommended therapies, such as ABVD (doxorubicin, bleomycin, vincristine, and dacarbazine) and escalated BEACOPP (escalated dosing regimen of bleomycin, etoposide, doxorubicin, cyclophosphamide, vincristine, procarbazine, and prednisone) [9–16].

In the USA, few studies have assessed the effects of caregiving on those who care for patients with cHL, particularly those caring for adult patients with cHL. Results from a cross-sectional study of caregivers for pediatric patients with lymphoma showed that nearly all caregivers (97%) reported low to moderate quality of life [17]. In a second study, which surveyed caregivers of children and adolescents with newly diagnosed high-risk cHL, results showed more disruption at work, as well as greater productivity loss, among caregivers whose children had lower health-related quality of life (HRQoL) [18].

As cHL has a bimodal age distribution, with one age peak in adolescents and young adults under 40 years and another > 70 years, many caregivers of patients with cHL would be projected to be of the age of the US workforce [19–22]. Given the importance of caregiving in cancer care and a lack of information on caregivers of adult patients with cHL, US–based caregivers were surveyed as part of the CONNECT (Classic Hodgkin Lymphoma: Real-World Observations From Physicians, Patients, and Caregivers on the Disease and Its Treatment) study—the first real-world survey in cHL to include patients, physicians, and caregivers—to better understand the burden and journey of those caring for adult patients with cHL. The survey also evaluated these caregivers’ views on treatment goals and on the roles they played in making treatment decisions. Additionally, as the study was conducted during the height of the COVID-19 pandemic, the impact of COVID-19 on caregiving was evaluated.

## Methods

### Study design

The CONNECT caregiver survey was an anonymous, 15-min online survey administered from December 30, 2020, to March 1, 2021. For this survey, a caregiver was defined as a person involved in managing a patient who was currently receiving or who previously received cHL treatment. Participants were informed of the study sponsor following survey completion; participant identities were blinded to the study sponsor and research organization. The survey was reviewed by the New England Institutional Review Board and granted exemption status.

### Caregiver recruitment

Caregivers in the USA were recruited using the following methods: (1) referral from patients completing the CONNECT patient survey and (2) direct caregiver recruitment. To increase reach, improve consistency, and minimize bias, caregivers were recruited using a multi-sourced recruitment model that included panels, referrals (snowball sampling), the Gryt Health Cancer Community,^1^ social media, non-profit organizations, and paid digital advertising. Because caregivers were recruited using multiple methodologies, the exact number of unique caregivers invited is unknown and response rates were not calculated.

Invitations for survey participation were sent via email to prospective participants across the USA. Eligible caregivers were assured that participation was voluntary, that the information provided was confidential, and that they could withdraw at any time. Caregivers provided their consent to be surveyed.

### Caregiver inclusion and exclusion criteria

Caregivers were screened at the beginning of the survey to ensure that study inclusion criteria were met.

Caregiver respondents were willing and able to participate in a 15-min online survey and self-identified as a current or former adult caregiver to a relative, spouse/partner, or friend with cHL by answering yes to the following question: “Have you ever been a caregiver for someone with classic Hodgkin lymphoma?” Eligible caregivers were aged ≥ 18 years at the time of the survey and directly involved in managing a patient with cHL who met the following patient criteria: aged ≥ 12 years at cHL diagnosis and ≥ 18 years at the time of the survey, diagnosed within the past 10 years, and diagnosed with stage I to stage IV cHL that was either currently being treated or was previously treated. Caregivers of patients who had died could participate if that death occurred within the 12 months prior to the survey.

Those self-identifying as professional or hired caregivers were excluded from participating; no other specific criteria were used to exclude caregivers from participating in the survey.

### Study survey

The CONNECT caregiver survey was developed based on constructs identified from the literature with input from clinicians and patient experience research experts from Gryt Health, and utilizing research industry best practices. It was piloted with 3 caregivers to ensure clarity, accuracy, and ease of understanding. The caregiver survey included questions on caregiver and cared-for patient demographics and clinical characteristics, the caregiving journey, the role of caregivers in making treatment decisions, and the effect of COVID-19 on caregiving. Information on caregiver age, sex, geographical location, employment status, and relationship with the cHL patient was obtained as part of the survey. The following information was obtained for the patient being cared for: age (at diagnosis and at time of survey), time since cHL diagnosis, disease stage at diagnosis, and disease stage at time of survey. Additionally, impacts of caregiving on HRQoL were evaluated using the PROMIS (Patient-Reported Outcomes Measurement Information System™)–Global-10 instrument [23, 24], and work productivity and impairment were evaluated using the modified 6-item Work Productivity and Activity Impairment Caregiver (WPAI:CG) questionnaire previously adapted for caregiving [25, 26]. The PROMIS Global-10 [23, 24] is a validated and publicly available global health assessment tool that measures the multi-dimensional construct of health and has been used across a wide variety of chronic diseases and conditions as well as for adults from the general population. The PROMIS Global-10 short form consists of 10 items that form two subscales of physical and mental health. The WPAI:CG [25, 26] was the first questionnaire developed to specifically measure caregivers’ assessment of the impact of caring for a chronically ill adult on work productivity and regular activity by quantifying the amount of time missed from work and the amount of reduced productivity while at work.

The CONNECT caregiver survey was conducted to understand the caregiver’s journey and views on how treatment goals are set and treatment decisions made. Caregivers were asked when they started caring for the patient and what the top treatment goals and considerations were for the caregiver and for the patient. Additionally, the survey assessed the caregiver’s understanding of and alignment with the patient’s treatment goals and identified who made key treatment decisions and discussed treatment options with the patient’s healthcare provider.

Lastly, the survey evaluated the impact of COVID-19 on caregiving. Participating caregivers were asked to indicate their level of agreement on a scale of 1 (completely disagree) to 5 (completely agree) with the following statements: (1) The COVID-19 pandemic impeded my ability to participate in the patient’s classic Hodgkin lymphoma care to the extent that I wanted to; (2) I was impacted by “no visitor” policies at the patient’s doctor appointments; (3) My job was impacted by COVID-19; (4) Because I care for an at-risk person, I limited my daily activities more than I would have to reduce my own risk of getting COVID-19.

### Study analysis

Analyses were conducted using WinCross and RStudio software. Univariate statistics were used to describe demographics and characteristics of caregiver respondents and the patients they cared for. Continuous data were reported as mean (SD) or median (range). Categorical data (e.g., level of education, location) were reported as individual totals and the respective percentages. For non–mutually exclusive categorical variables (e.g., treatments), individual totals and the respective percentage out of the total caregiver sample size are presented. Differences in responses across caregiver subgroups (spouse/partner and other caregivers, spouse/partner and parent caregivers, caregivers of patients on vs off treatment) were explored and compared statistically at a 95% confidence level. Continuous variables were analyzed using the Wilcoxon rank sum test, and categorical variables were analyzed using chi-square or Fisher’s exact tests.

### Data quality assurance

To ensure data quality, the survey incorporated skip logic so respondents were only asked relevant questions. Range checks were used to minimize erroneous responses that were outside the valid range (e.g., an age of “136” instead of “36” years) and were inconsistent with previous responses. A multi-level review process of test links ensured program accuracy. Data were checked for quality by evaluating completion times and evidence of selection of multiple unrealistic, internally inconsistent responses. Missing data were anticipated to be minimal, as respondents did not have the option to not respond for most questions. Any responses that were unknown (including missing values) were not utilized in statistical analyses.

## Results

### Caregiver characteristics

**Fig. 1:**
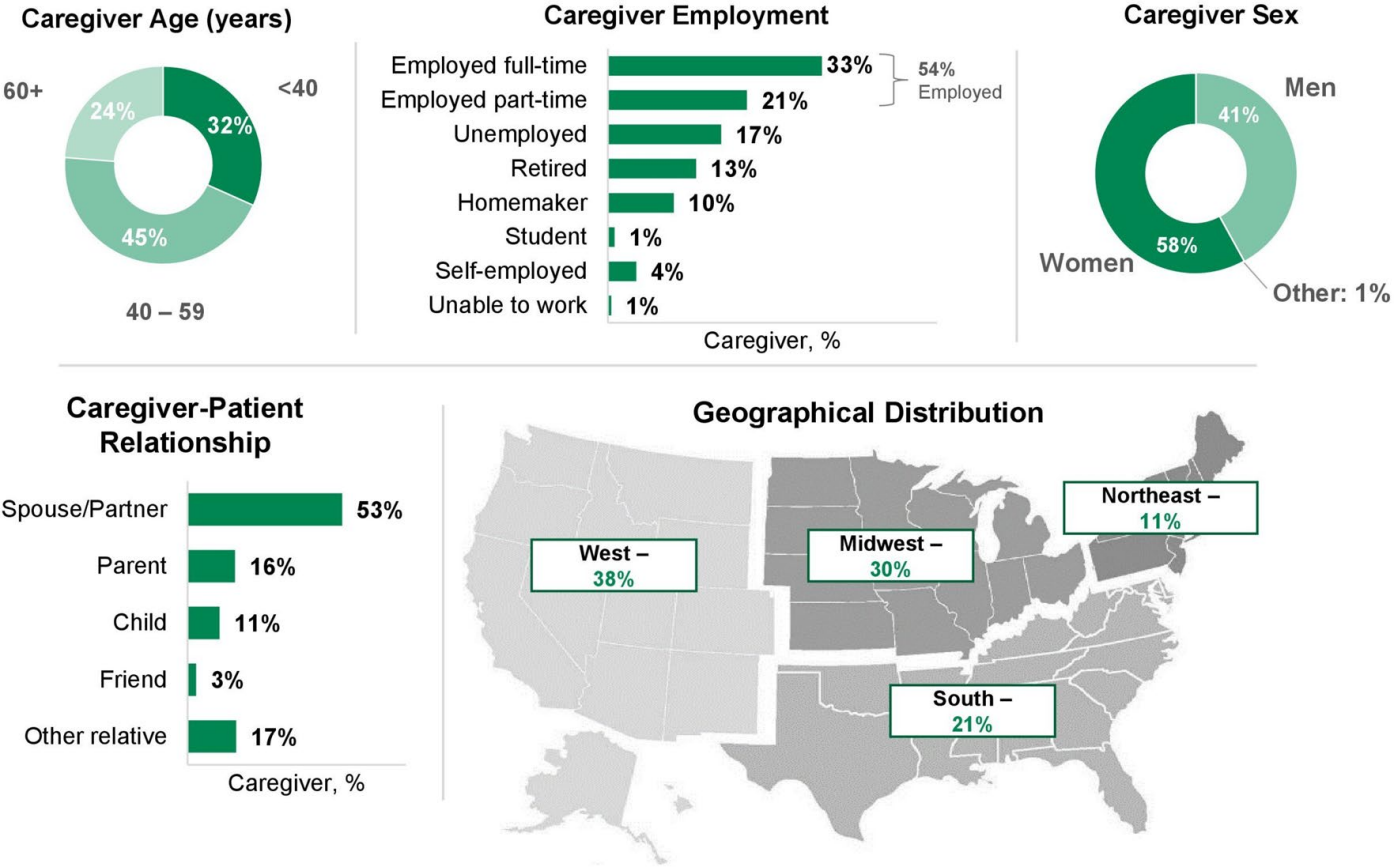
Caregiver characteristics (*N* = 209)

The survey was completed by 209 caregivers; 53% were the spouse/partner of a patient with cHL (Fig. 1). Caregivers’ median age (IQR) was 47 years (36–59) (spouse/partner caregivers, 47 years (38–59); other caregivers, 48 years (35–59)), with 77% of caregivers aged less than 60 years. Overall, 58% of caregivers were female (spouse/partner caregivers, 54%; other caregivers, 63%). Fifty-four percent of caregivers were employed full- or part-time at the time of survey completion (spouse/partner caregivers, 56%; other caregivers, 51%), and 4% were self-employed (spouse/partner caregivers, 3.6%; other caregivers, 5.1%). In total, 68% of caregivers were from the West or Midwest regions of the USA (spouse/partner caregivers, 74%; other caregivers, 62%).

### Patient characteristics

**Fig. 2:**
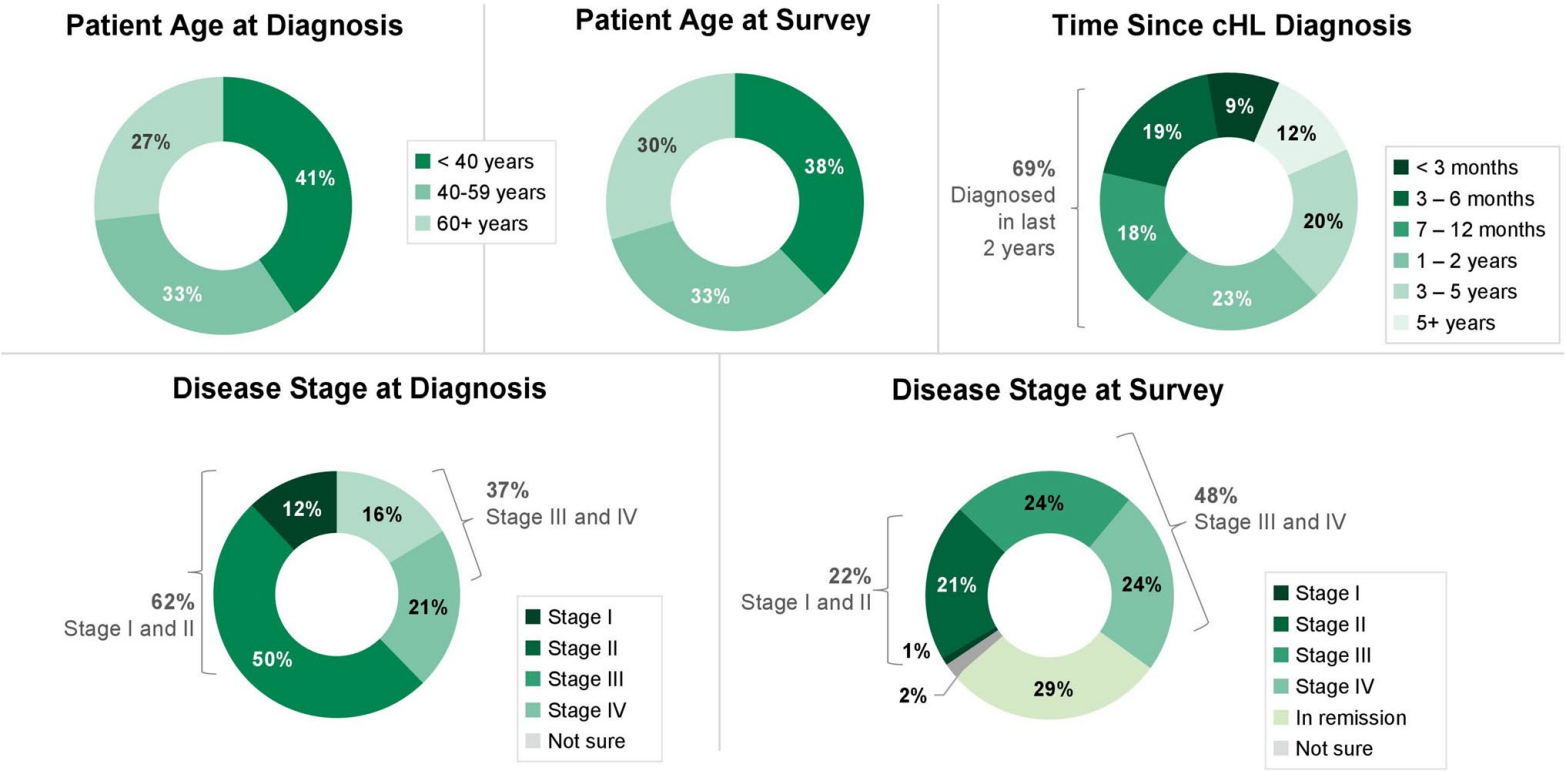
Characteristics of patients cared for by participating caregivers. *cHL* classic Hodgkin lymphoma

Sixty-nine percent of patients cared for were diagnosed with cHL within the prior 2 years (Fig. 2). Seventy-four percent of patients were aged less than 60 years at cHL diagnosis, and 71% of patients were aged less than 60 years at the time of the survey. Thirty-seven percent of patients were initially diagnosed with stage III or IV cHL. Fifty-eight percent of patients being cared for had previously received but were not actively receiving treatment, and 42% were actively receiving treatment at the time of the survey.

### Impact of caregiving on quality of life and work

Mean summary subscale scores from the PROMIS-Global on caregivers’ HRQoL were similar to US normative data for both physical and mental health (Table 1)*.* While physical health scores did not differ from population-level mean values, these scores were significantly lower for parent caregivers compared with spouse/partner caregivers (mean T-score: 48.5 vs 52.7; *P* = 0.007). Of the 117 employed caregivers responding to the 6-item WPAI:CG questionnaire, caregivers of patients on vs off treatment reported greater productivity burdens, especially for absenteeism (*P* = 0.020) and work impairment (*P* = 0.054), as measured by the WPAI:CG. There were no significant differences by employed caregiver relationship, including spouse/partner caregivers vs parent caregivers.

**Table 1:** cHL informal caregiver quality of life and work impairment

	Total	Patient on treatment	Patient off treatment	*P-*value^a^	Spouse/partner	Other relationship	*P-*value^b^	Parent	*P-*value^c^
PROMIS-Global 10, mean T-score (SD)^d^	*N* = 209	*n* = 88	*n* = 121		*n* = 111	*n* = 98		*n* = 34	
Physical health^e^	52.1 (7.1)	53.5 (7.2)	51.1 (6.9)	0.017	52.7 (6.3)	51.4 (8.0)	0.211	48.5 (7.9)	0.007
Mental health^f^	49.6 (7.9)	49.1 (8.5)	49.9 (7.5)	0.465	48.8 (7.3)	50.5 (8.5)	0.107	46.1 (7.2)	0.070
WPAI:CG, %^g^	*n* = 117	*n* = 47	*n* = 70		*n* = 63	*n* = 54		*n* = 17	
Absenteeism	11	18	6	0.020	6	16	0.060	14	0.376
Presenteeism	21	23	20	0.458	23	18	0.342	18	0.444
Work impairment	29	36	24	0.054	29	28	0.884	28	0.881
Activity impairment	22	24	20	0.409	21	22	0.878	17	0.509

^a^Comparison for on treatment vs off treatment

^b^Comparison for spouse/partner vs other relationship

^c^Comparison for spouse/partner vs parent

^d^Score interpretation: poor, 20 to < 30; fair, 30 to < 40; good, 40 to < 50; very good, 50 to < 60; excellent, 60 to 80 [23, 24]

^e^PROMIS Scale v1.2 Global Health—Physical Health 2a: sum the values of the responses to item Global03 and Global06 and convert to *T*-scores

^f^PROMIS Scale v1.2 Global Health—Mental Health 2a: sum the values of the responses to item Global04 and Global05 and convert to *T*-scores

^g^Total percentage of time work/activity affected among employed caregivers [25, 26]

*cHL* classic Hodgkin lymphoma, *PROMIS T-score* Patient-Reported Outcomes Measurement Information System® Transformed scores of 50 (SD, 10), *SD* standard deviation, *WPAI:CG* Work Productivity and Activity Impairment: as adapted for caregiving

### Caregiver journey

**Fig. 3:**
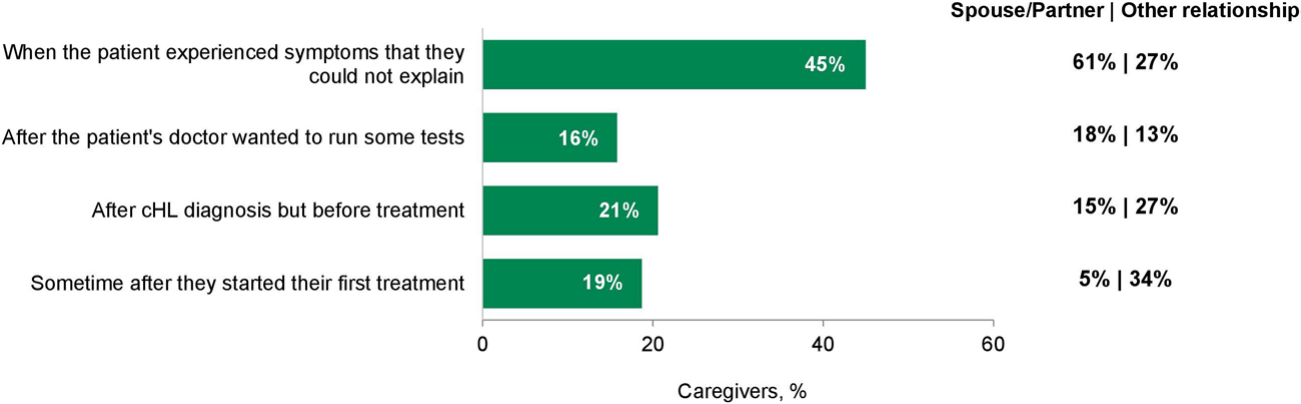
Caregiver journey: when caregiver started caring for the patient. Caregivers were asked when they started caregiving for the patient and selected the best answer from those provided. Total caregivers, *N* = 209; spouse/partner, *n* = 111; other relationship, *n* = 98. *cHL* classic Hodgkin lymphoma

When caregivers were asked when they started caring for the patient, responses differed significantly between spouse/partner caregivers and other caregivers (*P* < 0.001). Caregiving started at an earlier point in the patient’s journey for most spouse/partner caregivers compared with other caregivers (Fig. 3). More spouse/partner caregivers than other caregivers began caregiving at the onset of patient symptoms (61% vs 27%). More other caregivers than spouse/partner caregivers began caregiving after the patient was diagnosed with cHL, but before treatment (27% vs 15%) or after the patient’s first treatment (34% vs 5%).

### Caregiver role in treatment decisions

**Fig. 4:**
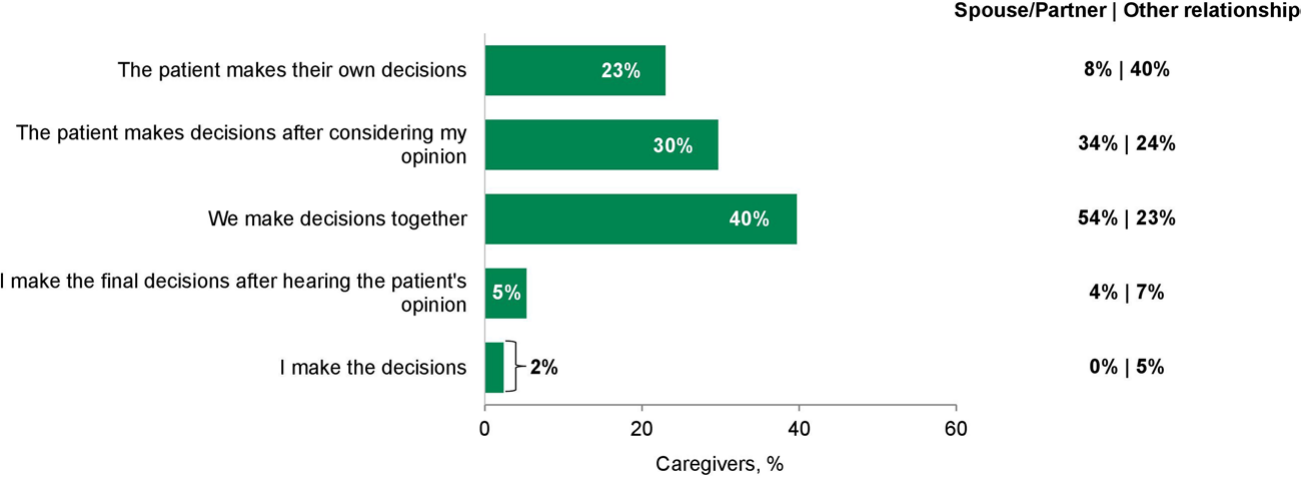
Treatment decision-making. Participants were asked how they and the person they care for make key decisions about treatment and selected the best response from those provided. Total participants, *N* = 209; spouse/partner, *n* = 111; other relationship, *n* = 98

When caregivers were asked how they and the patient they cared for make key decisions about treatment, responses differed significantly between spouse/partner caregivers and other caregivers (*P* < 0.001). Eighty-eight percent of spouse/partner caregivers and 47% of other caregivers reported discussing treatment options together with the patient. Forty percent of caregivers reported making treatment decisions jointly with the patient (Fig. 4), with more spouse/partner caregivers reporting making treatment decisions jointly compared with other caregivers (54% vs 23%).

**Fig. 5:**
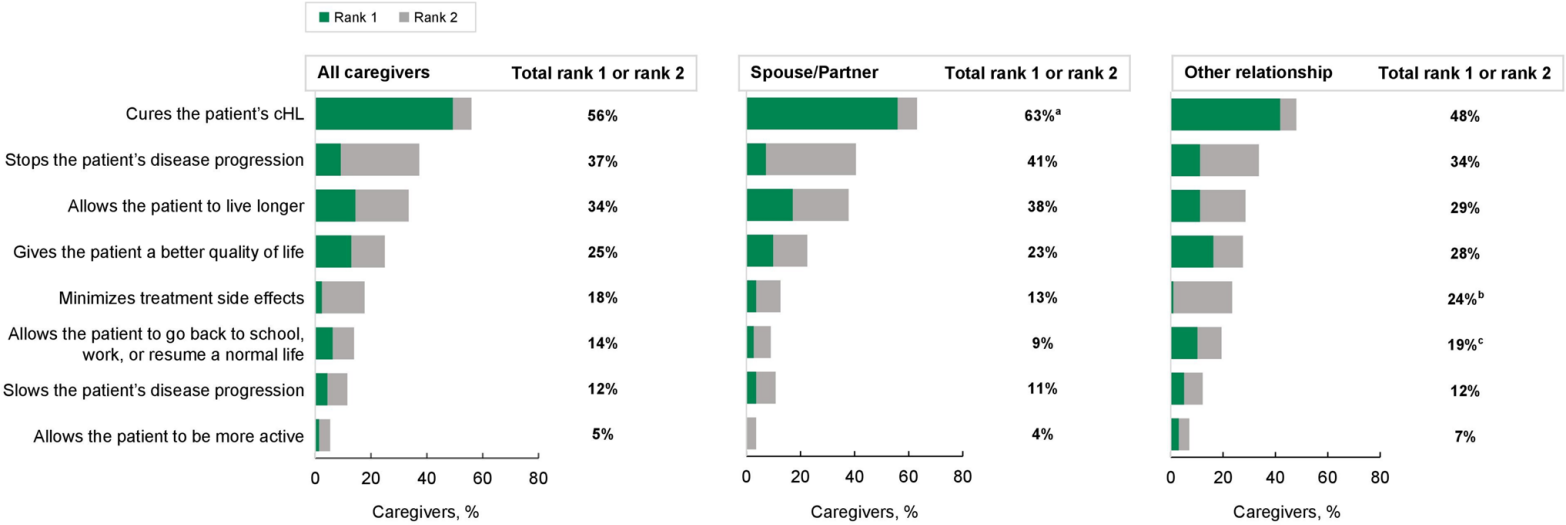
Caregiver goals for cHL treatment. Participants were asked to rank their top treatment goals and considerations when thinking about a patient’s cHL treatment. Total participants, *N* = 209; spouse/partner, *n* = 111; other relationship, *n* = 98. ^a^A significant difference (*P* = 0.028) between spouse/partner and other relationship. ^b^A significant difference (*P* = 0.040) between spouse/partner and other relationship. ^c^A significant difference (*P* = 0.030) between spouse/partner and other relationship. *cHL* classic Hodgkin lymphoma

Caregivers’ top treatment goals (rank of 1 or 2) for the patients they cared for were curing cHL, stopping disease progression, and extending the patient’s life (Fig. 5). When asked about treatment goals, 56% of caregivers ranked cure either first or second (rank of 1 or 2), with 49% of caregivers specifically ranking cure first (rank of 1). Significantly, more spouse/partner caregivers than other caregivers ranked cure as their top treatment goal (rank of 1 or 2; 63% vs 48%; *P* = 0.028).

Spouse/partner caregivers compared with other caregivers more frequently participated in discussions with physicians about treatment options (52% vs 28%) and took part in treatment decisions with the patient (54% vs 23%). Compared with other caregivers, spouse/partner caregivers had more extensive conversations with the person they cared for about treatment options (88% vs 68%), were more frequently aligned with the patient’s treatment goals (93% vs 79%), felt physician conversations regarding side effect management were important (94% vs 84%), and thought that the doctor provided adequate side effect information (91% vs 71%).

### COVID-19 impacts

Caregivers reported COVID-19 impacts, such as having to limit daily activities to reduce COVID-19 risks (72%), “no visitor” policies for doctor appointments (52%), and limitations on the extent to which they were able to participate in their patient’s cHL care (34%).

## Discussion

The CONNECT caregiver survey was conducted to highlight the burden of caregiving for adult patients with cHL, particularly for spouse/partner caregivers, who made up the majority of study participants. Although this study did not identify decreased HRQoL for caregivers compared with the general US population, it did find that caregiving negatively impacts work productivity. Additionally, data also showed that patients and caregivers have similar treatment goals, and that caregivers are involved in treatment discussions. As this survey was conducted during the height of the COVID-19 pandemic, responses indicated that the COVID-19 pandemic did impact caregiving activities, including limiting their daily activities and their ability to participate in the patient’s care.

Caregiver participants in the CONNECT survey were generally similar to those who participated in a National Survey of Cancer Caregivers performed over a similar timeline (February 2021–July 2021) [2]. In the National Survey of 2703 caregivers, participants were slightly older with a similar percentage of female participants. Although more caregivers reported working full-time in the National Survey, full-time work was defined as 30 or more hours per week. More spouse/partner caregivers participated in the CONNECT survey than in the National Survey (53% vs 12%), perhaps reflecting the bimodal age distribution of patients diagnosed with cHL.

Results from the CONNECT survey, the first known study to describe the burden that caregivers of adults with cHL experience, show that regardless of their relationship with the patient, caregivers on average reported physical and mental health similar to that of the general US population [23, 24]. However, caregivers did report negative impacts on work productivity. Spouse/partner caregivers were slightly more impaired (i.e., more presenteeism) during work due to caregiving, whereas non-spouse/partner caregivers were more likely to miss work (i.e., more absenteeism). Among working caregivers, those caring for a patient on treatment experienced a greater productivity impact, particularly in absenteeism and work impairment, vs caregivers of patients off treatment. While there is limited research available regarding caregivers of adult patients with cHL in the USA, a negative impact on work productivity with cancer treatment was found in a systematic literature review conducted to examine how pharmacologic treatment of any cancer affected work productivity in patients and their caregivers, with studies most commonly using the WPAI questionnaire (*n* = 9) [27]. The review found that intensive cancer treatment was the most common contributing factor to productivity impairment; other factors reportedly associated with impairment were disease progression and severity, cognitive/neurological impairments, poor health status, and receipt of chemotherapy.

Two other studies that evaluated caregiver burden and work productivity using the WPAI questionnaire were identified. A population-based study quantifying burden of caregiving for patients with cancer in Europe found that caregivers (*n* = 1713) were 1.75 times more likely to experience absenteeism compared with non-caregivers (*n* = 103,868), with a mean WPAI score of 8.97 compared with 5.13 (*P* < 0.05) [28]. Caregivers were 1.46 times more likely to experience overall work impairment, with a mean WPAI score of 28.67 compared with 18.62 (*P* < 0.05). Another study of employed caregivers (*n* = 39) of patients with advanced-stage cancers reported a mean loss in work time (absenteeism) of 10% and a work productivity loss of 23% over 15 months; a significant increase in overall work productivity loss was observed, particularly in spouse caregivers [25]. Treatments, including those given in the frontline setting, that improve survival and decrease the need for subsequent therapies may lighten caregiver burden by lessening absenteeism and work impairment.

Since the Institute of Medicine included patient-centered care as an essential characteristic of quality healthcare in 2001 [29], the model of clinical decision-making has shifted. Where once decision-making was controlled by clinicians, now it is a shared process that is patient led and patient centered, with many patients choosing to involve a caregiver in treatment decision-making [2]. We found that caregivers participating in the CONNECT survey play an important role in treatment decisions, with 40% of caregivers surveyed participating in decision-making with the patient. Specifically, these caregivers reported extensively discussing treatment options with the patient. Results from the CONNECT patient survey show that 94% of patients reported having a caregiver, and 28% of patients reported that their caregiver is involved in discussions with the physician about treatment options [30]. Although not specific to cHL, results from a national survey of more than 2700 cancer caregivers were similar, with 42% of caregivers reporting being involved in deciding whether to begin treatment and 50% of caregivers reporting being involved in deciding the treatment plan [2].

Several similarities were found between survey results for all patients and caregivers who participated in CONNECT [30]. First, both patients and caregivers ranked cure (72% and 56%) and stopping disease progression (46% and 37%) the highest when asked to rank their top 2 treatment goals. Other treatment goals that 20% of patients or caregivers ranked as either their first or second treatment goal were to live longer (33% and 34%) and to have a better quality of life (14% and 25%). Second, both patients and caregivers felt that the health provider informed them about side effects, with 80% of patients reporting being informed about short-term effects and 70% reporting being informed about long-term effects. The CONNECT caregiver survey did not differentiate between short- and long-term effects as did the patient survey, but the majority of caregivers thought that doctors provided adequate side effect information.

### Limitations

Results from this survey may not be generalizable to all informal caregivers of patients with cHL due to the nature of convenience sampling by way of an opt-in group of surveyed participants and the need for participants to be able to access an online platform. While caregivers were asked about their workforce participation at the time of the survey, they were not specifically asked how caregiving had resulted in changes in their workforce participation at the time of diagnosis and/or survey administration. Lastly, we did not determine if participants recruited via different mechanisms were systematically different from one another or verify that respondents who self-identified as caregivers were in fact caregivers of patients with cHL.

## Conclusions

In this study, 53% of caregivers of adults with cHL were spouses/partners. Although cHL patient caregivers reported HRQoL similar to that of the general population, caregiving impacted their work productivity regardless of their relationship to the patient. Spouse/partner caregivers vs other caregivers were more involved, were involved earlier, and reported greater alignment with patient treatment goals and decision-making. Cure was caregivers’ top treatment goal. While all caregivers played an active role in the patient’s journey, spouse/partner caregivers were more involved in treatment plans and decision-making.

## Footnotes

1 The Gryt Health Cancer Community is an online community of cancer patients, survivors, and caregivers; Gryt Health supports this community through connection, education, and advocacy.
